# The Effect of an Active Plant-Based System on Perceived Air Pollution

**DOI:** 10.3390/ijerph18158233

**Published:** 2021-08-03

**Authors:** Tatiana Armijos Moya, Marc Ottelé, Andy van den Dobbelsteen, Philomena M. Bluyssen

**Affiliations:** 1 Faculty of Architecture and the Built Environment, Delft University of Technology, 2628 BL Delft, The Netherlands; A.A.J.F.vandenDobbelsteen@tudelft.nl (A.v.d.D.); P.M.Bluyssen@tudelft.nl (P.M.B.); 2 Faculty of Civil Engineering & Geosciences, Delft University of Technology, 2628 CN Delft, The Netherlands; M.Ottele@tudelft.nl

**Keywords:** plant monitoring, indoor air quality, pollution sources, sensory assessment, active plant-based system, phytoremediation

## Abstract

Active plant-based systems are emerging technologies that aim to improve indoor air quality (IAQ). A person’s olfactory system is able to recognize the perceived odor intensity of various materials relatively well, and in many cases, the nose seems to be a better perceiver of pollutants than some equipment is. The aim of this study was to assess the odor coming out of two different test chambers in the SenseLab, where the participants were asked to evaluate blindly the level of acceptability, intensity, odor recognition, and preference at individual level with their noses. Two chambers were furnished with the same amount of new flooring material, and one of the chambers, Chamber A, also included an active plant-based system. The results showed that in general, the level of odor intensity was lower in Chamber B than in Chamber A, the level of acceptability was lower in Chamber A than in Chamber B, and the participants identified similar sources in both chambers. Finally, the preference was slightly higher for Chamber B over Chamber A. When people do not see the interior details of a room and have to rely on olfactory perception, they prefer a room without plants.

## 1. Introduction

The improvement of indoor air quality (IAQ) is a constant and significant concern among researchers. Indoor air pollutants can be emitted by several indoor sources, such as furnishings, building materials, and cleaning products [1,2,3,4,5]. Additionally, plants, which are generally not mentioned in the list of pollutant sources, can be, according to several findings, a source that can pollute as well as clean the air [6]. Furthermore plants have the potential to generate attractive and friendly environments that support social and emotional wellbeing [7,8]. Numerous studies have described, evaluated, and analyzed the effect of passive plants on cleaning the indoor air [9,10,11,12,13,14]. In a previous study, in which the air cleaning effect of formaldehyde by potted plants was tested in a laboratory study, it was concluded that to meet the minimum ventilation rates in breathing zones, it is necessary to introduce at least 36–42 plants for every square meter of floor space [15]. In that study, formaldehyde was introduced in a glass chamber in which the plant and its substrate were located, hereby surrounding the plant and its substrate with formaldehyde. In a “normal” indoor environment, usually the source of formaldehyde is not close to the plant system, and therefore, for the plant system to take-up the formaldehyde, the polluted air needs to be transported to the vicinity of the plant. This can, for example, be realized by an active plant-substrate system, in which the contaminated air is forced to go through the plant leaves and through the substrate roots.

On the other hand, health and comfort complaints have been associated with poor IAQ due to emissions from building materials and furnishings where people spend most of their time. These complaints can range from annoying smells to symptoms such as dry eyes, irritated skin, and airway problems, to carcinogenic effects [16,17]. These problems have been linked to sick building syndrome (SBS) (biological or psychological problems caused by the negative impact of buildings) [18], or building-related illness (BRI) [4]. Odors may cause a variety of undesirable effects in users, fluctuating from annoyance and discomfort to acknowledged health and psychological stresses [4,19]. In the indoor environment, odorous VOCs (volatile organic compounds) are emitted from several construction and cleaning products [20,21,22]. In most situations, these emissions are present in very low concentrations that are difficult to measure chemically, but are perceived by the users. The human nose is capable of detecting certain odorous pollutants at ppm level and sometimes even at ppb level, while most chemical instruments monitor at ppm level [23,24]. However, there are also pollutants that cannot be assessed by people, but are still important to assess because of their toxic effects. Then, Yi et al. (2013) reported that indoor pollutants with the highest chemical concentrations were not the most odor-active odorants [25]. It is therefore important to assess IAQ both by chemical instruments (chemical air pollution) and with the nose (sensory air pollution). Sensory assessment with people (trained or untrained) [20] has been used to assess indoor air quality in offices and the pollution emitted from different building and furnishing materials in several laboratory studies [1,26].

The aim of the study was to test the effect of an active plant-based system on the perceived air quality of a recently furnished room. For this purpose, two identical test chambers of the SenseLab were furnished with new carpet tiles. In one of the chambers, an active plant based-system was added. The SenseLab is a laboratory for testing and experiencing single and combinations of indoor environmental conditions located at Delft University of Technology [27].

## 2. Materials and Methods

### 2.1. General

Based on previous studies, first an active plant system was designed and realized. Then, two test chambers of the SenseLab [27] were furnished identically, and in one of the test chambers, the active plant system was installed. Then, the test chambers were made ready for perception of the air by people without entering the chambers. Firstly, a pilot test was held to evaluate the setup with 59 participants, followed by three sessions in April and May 2019, in which the untrained participants visited the SenseLab to participate in a “sniffing test” to evaluate the air from the two test chambers (Table 1).

### 2.2. Active Plant-Based System

From earlier conducted experiments on the depletion of formaldehyde by potted plants [15], it was concluded that the effect of potted plants on “cleaning” indoor air is limited. It was suggested to investigate whether an active plant system could give better results. In addition, it was found that (a) the growth medium has a big influence in the depletion of formaldehyde and (b) the plant *Nephrolepis exaltata L*. in the medium expanded clay performed the best.

Therefore, for the prototype of the active plant-based system, 30 *Nephrolepis exaltata L.* (also known as Boston Fern) plants were re-potted on expanded clay. In addition to having the best performance in the depletion of formaldehyde during the laboratory tests, the density of this medium allows the air to go through the substrate easier than it does with other substrates (e.g., soil). The structure of the active plant-based system was built with low-emitting materials: aluminum frame together with Plexiglas elements that held the 30 plants.

**Figure 1:**
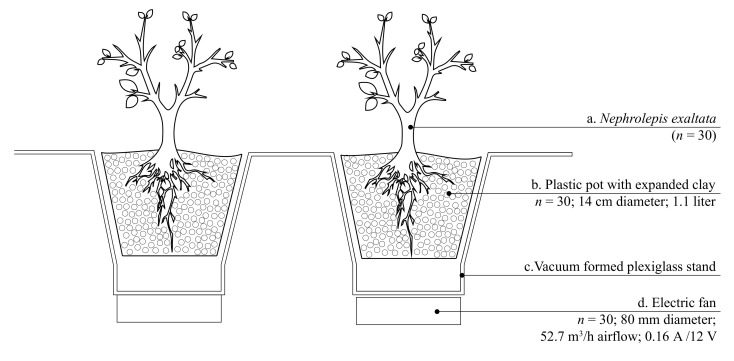
Prototype section scheme of the active plant-based system.

Each of the 30 plants was then connected to a fan (80 mm diameter, airflow: 52.7 m^3^/h, 0.16 A/12 V, 1.92 watt, sound level: 0.3 sone/22.4 dB) that forced the air to go through the plant itself and through the growth medium. To keep the plants in the system alive and provide the right amount of light that the plants need to grow, a growing LED (Light-emitting diode) light (0.31 m × 0.31 m × 0.035 m, 120 W, number of LED lamps: 1365 = 1131 red and 234 blue, wavelength red: 630–660nm, wavelength blue: 430–450nm, maximum surface illumination: 2–3 m^2^, 2506 lux at 1.5 m distance from the lamp) was installed in Chamber A. The fans were activated for 12 hours every day during the whole experiment with the help of a plug-in mechanical timer, and the plants received water once per week. Figure 1 presents a section of the prototype. As mentioned before, the active plant-based system was activated for 12 hours per day, resulting in a total energy consumption of 778 kWh per year (252 kWh for the fans + 526 kWh for the growing LED light).

### 2.3. The SenseLab and the Test Chambers

**Figure 2:**
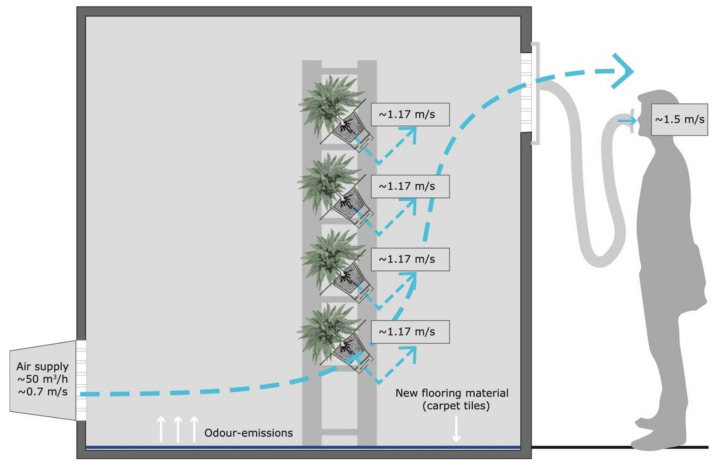
Setup of Chamber A (including an active plant-based system).

**Figure 3:**
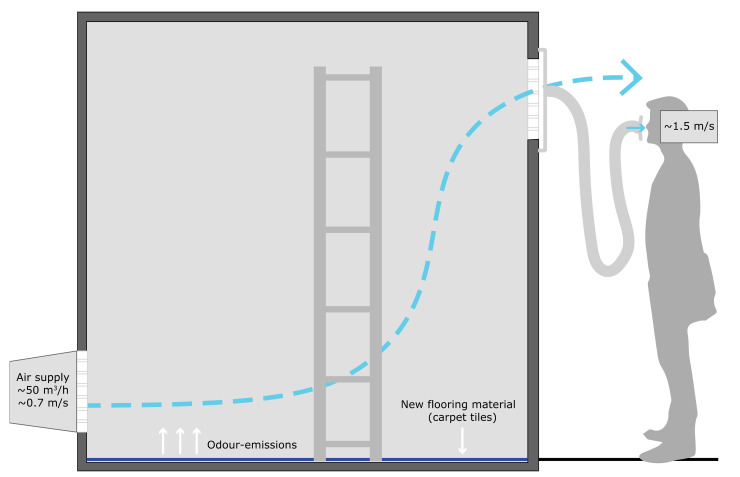
Setup of Chamber B.

To evaluate the efficacy of the active plant-based system on the perceived air quality, two chambers in the SenseLab were selected to execute the experiment. The two test chambers have the same features and are constructed of low-emitting materials to ensure a good air quality. Each chamber, having a volume of 19.7 m^3^ (floor area 9.36 m^2^ × 2.1 m height), was furnished with 9.36 m^2^ of new carpet tiles. Figure 2 and Figure 3 illustrate the selected setup for each chamber. In Chamber A, an active plant-based system was placed to evaluate the effect of that system on the (perceived) air quality (Figure 2). A 110 mm diameter flexible air duct was connected to the air outlet of each chamber to allow the participants to evaluate the air coming out of the chamber, without going into the chambers (and thus not seeing what they were assessing) (Figure 2 and Figure 3).

The temperature (T), relative humidity (RH), and CO_2_ levels were monitored during the whole experiment (from 04 April 2019 to 14 May 2019). Three sessions of evaluation with subjects were executed every two weeks. The sessions were executed during the same season around the same time to keep the variables as similar as possible. The changes in temperature and relative humidity inside of the chambers were monitored with two HOBO External data loggers, and the CO_2_ levels were monitored with HOBO^®^ MX CO_2_ loggers. A FlowFinder MK2 and ComfortSense Mini (compact anemometer for ventilation and draught measurements) were used to measure the air supply in the chambers and the air velocity in the (sniffing) tube, respectively. Additionally, a VOC-monitoring instrument (Photoionisation Detector (PID) ppbRAE 3000 with 11.7 eV UV lamp) was used to measure the air pollutants coming out of both chambers. It is important to mention that all the instruments used during this study were calibrated and tested prior the experiments to obtain reliable data.

### 2.4. The Questionnaire

**Figure 4:**
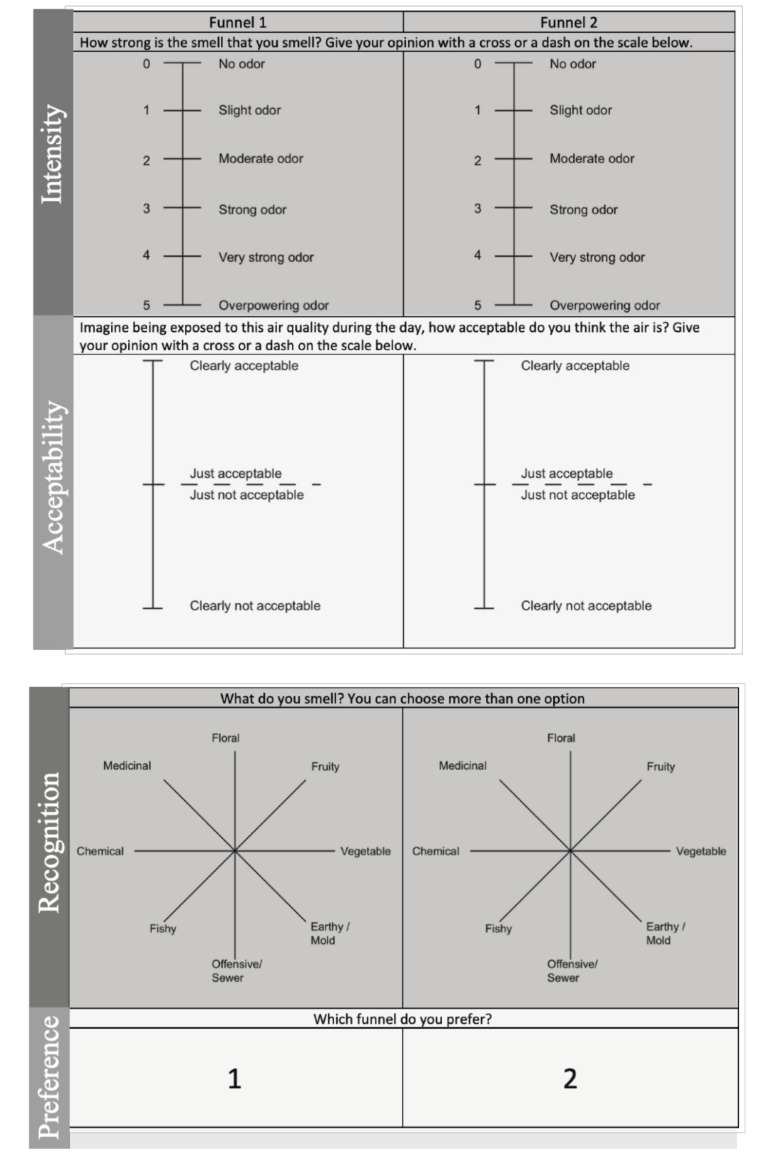
Questionnaire: intensity, acceptability, odor recognition, and preference assessments.

To assess the perceived air quality coming out of the chambers, a questionnaire was developed based on the questionnaire used by Gunnarsen and Fanger, 1992 [28]. For this study, intensity, acceptability, recognition of the odor, and preference were assessed (Figure 4). The participants were asked the following questions: “How strong is the odor that you smell? Give your opinion with a cross or a dash on the scale below (intensity)”; “Imagine being exposed to this odor during the day, how acceptable do you think this odor is? Give your opinion with a cross or a dash on the scale below (acceptability)”; “What do you smell? You can choose more than one option (odor recognition)”; “Which funnel do you prefer? (preference)”. Regarding intensity, the perceived odor was assessed on a five-grade scale. The acceptability level was assessed on a three-grade scale. The preference was assessed on an eight-point roulette where multiple choices were allowed. Finally, the participants had to choose which chamber they preferred.

### 2.5. Pilot Test

To evaluate the procedure and the questionnaire, a pilot test composed of three sections—intensity, acceptability and recognition—was executed on 4 April 2019 with 59 participants. Before the participants completed the questionnaire, an explanation was given of the contents and purpose of the experiment and questionnaire. In general, it took the participants approximately 3–5 min to perform the test and complete the questionnaire. After the pilot test, the questionnaire was modified and the section “Preference” was added in the last part of the questionnaire. Additionally, the air supply rate to all the test chambers (four in total) was changed from 1000 m^3^/h to 500 m^3^/h to reduce the amount of air (~130 m^3^/h to ~50 m^3^/h inside of the selected chambers); therefore, the air velocity also changed from 2.3 m/s to 1.5 m/s coming out of each funnel. During the pilot test, the air velocity was found to be too high (2.3 m/s) for properly assessing the perceived air quality. It is important to mention that every funnel had a diameter of 0.11 m, and with 1.5 m/s air velocity, therefore, 12 L/s of air were coming out of the funnel.

### 2.6. Procedure

After the pilot test, the questionnaire was fixed and three sessions of assessments were performed, each with two weeks in between. The recruitment of the participants was on a voluntary basis. When the participants arrived in the SenseLab, they were given a questionnaire with some general information about themselves and with some questions related with the assessment of the odor coming out of every chamber. The participants were assigned randomly where to start the assessment (e.g., Chamber A or Chamber B). The participants were asked to give their assessments of the air for each of the chambers by completing the questionnaire. For each question, the participants were allowed to take only one sniff in order to avoid “adaptation” to the smell coming out of the funnel [28]. After they finished the questions of the first chamber, they were asked to continue with the second chamber, and follow exactly the same procedure.

The participants took 5 min to perform the assessment and complete the questionnaire of both chambers.

### 2.7. Ethical Aspects

The Ethics committee of the TU Delft provided approval for the study. Each participant received a voucher after they completed and handed in their completed questionnaire. By handing in the questionnaire, they gave consent for using their assessments.

### 2.8. Data Management and Analysis

All data from the questionnaires were manually typed in and stored in IBM SPSS Statistics version 25.0 and Microsoft Excel. First, comparisons of mean values were performed with independent t-tests, and standard deviation and standard errors were used to evaluate levels of intensity and acceptability of every chamber. These tests were used to evaluate whether statistically significant differences between different sessions and chambers occurred, in time. In addition, descriptive statistical analyses were executed to evaluate frequencies in odor recognition and preference, and chi-square tests were executed to evaluate the correlation between the starting funnel and the level of preference of the participants.

## 3. Results

### 3.1. VOC, Temperature, Relative Humidity, and CO_2_ Monitoring

Although the participants pointed out that they perceived an odor (in all of the sessions and for both chambers), the 11.7 eV PID monitor used to measure the TVOC concentration resulted in 0 ppb for both chambers. This could be due to the measurement range of the VOCs that the instrument can detect, or the detection limit of the instrument (range: 0.01 ppm to 2000 ppm; resolution: 10 ppb).

**Figure 5:**
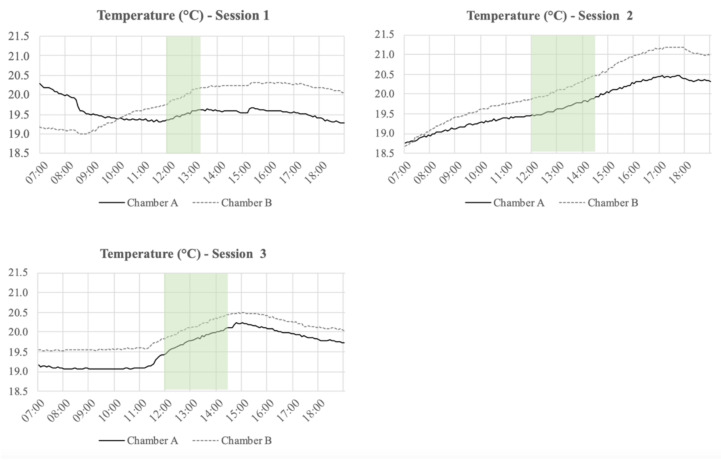
Temperature (°C) measured during the three sessions from 7.00 to 19.00 every 5 min. The highlighted section is the period when the participants executed the experiment and filled the questionnaires.

**Figure 6:**
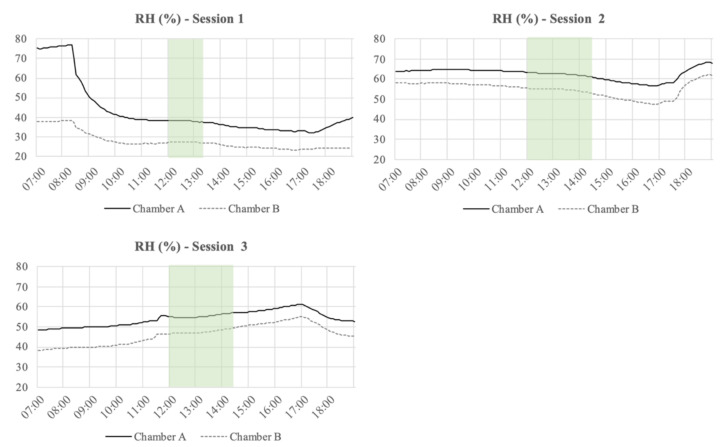
Relative humidity (RH) (%) measured during the three sessions from 7.00 to 19.00 every 5 min. The highlighted section is the period when the participants executed the experiment and filled the questionnaires.

**Figure 7:**
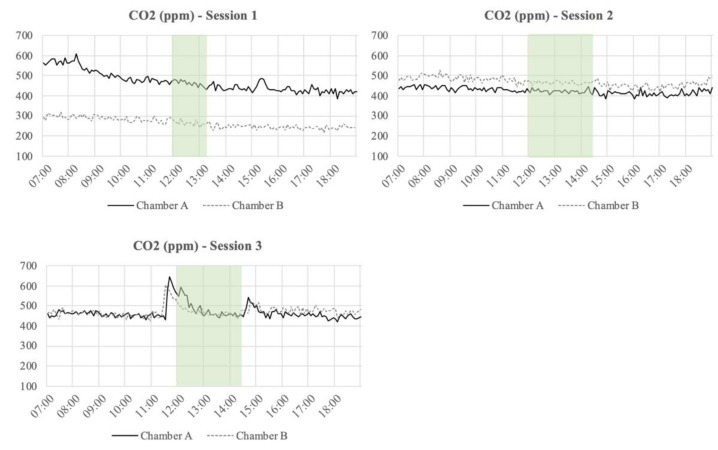
CO_2_ levels (ppm) measured during the three sessions from 7.00 to 19.00 every 5 min. The highlighted section is the period when the participants executed the experiment and filled the questionnaires.

The temperature, relative humidity (RH), and CO_2_ concentration inside of the Chambers A and B at each of the session days are presented in Figure 5, Figure 6 and Figure 7, respectively. The figures also include the period where the participants executed the experiment and filled in the questionnaires. Moreover, Table 2 presents the average temperature, RH, and CO2 concentration inside of the Chambers A and B during the execution of the sessions. From the measurements, it can be seen that the RH levels were always higher in Chamber A than in Chamber B. In general, the temperature levels inside of the Chamber A were always lower than in Chamber B. In Chamber A, with the active plant-based prototype, the CO_2_ levels were on average ~197 ppm higher than in Chamber B during the first session. Table 2 shows that the CO_2_ levels in Chamber B during session 2 and 3 were quite similar, while in Chamber A, those levels presented a slight fluctuation. Finally, the air supply and the air velocity of the chambers were measured before the sessions (air supply: ~50 m^3^/h; air velocity: ~0.7 m/s) and stayed constant throughout the execution of the experiment.

### 3.2. Intensity

**Figure 8:**
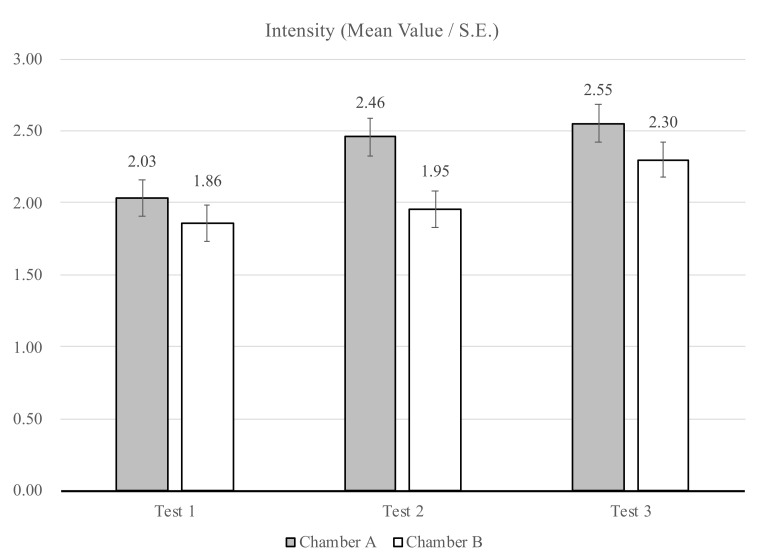
Intensity assessment for the two chambers at the three sessions: mean values and standard errors (SEs) (Session 1: *n* = 44; Session 2: *n* = 57; Session 3: *n* = 46).

The participants were asked to take a sniff from one of the two funnels and to answer the following question: “How strong is the odor that you smell? Give your opinion with a cross or a dash on the scale below (intensity)”. Table 3 presents the mean values of the intensity assessment of Chambers A and B together with standard deviations (SDs) and standard errors (SEs). From Figure 8, it can be seen that during the three sessions of assessment, in general, the participants evaluated the odor of Chamber A (in funnel A) as stronger than the odor of Chamber B (in funnel B).

Several independent-samples t-tests were conducted to evaluate whether differences between the odor intensity assessments of Chambers A and B were present during the sessions, and between the sessions. The analysis resulted in a statistically significant difference in odor intensity between the two chambers for Session 2 (*p* = 0.004). Additionally, a statistically significant difference between Session 1 and Session 2 (*p* = 0.02), and between Session 1 and Session 3 (*p* = 0.005), was found for Chamber A, indicating the odor intensity became stronger over time. For Chamber B, a statistically significant difference between Session 1 and Session 3 (*p* = 0.022) was found, showing that the odor intensity in Session 1 was significantly lower than in Session 3.

### 3.3. Acceptability

**Figure 9:**
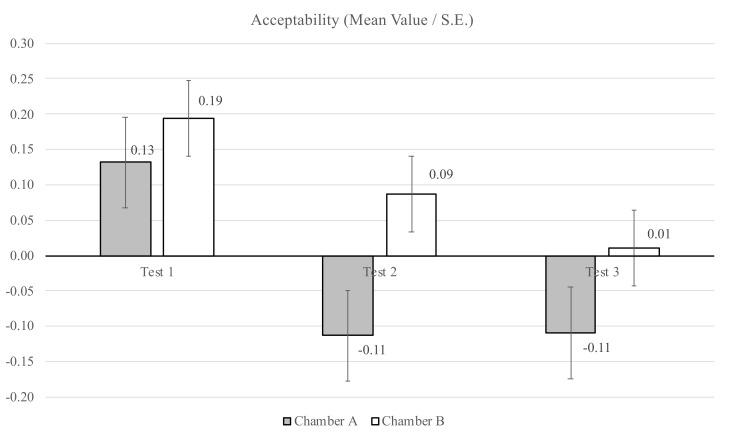
Acceptability assessment for the two chambers during the three sessions: mean values and standard errors (SEs) (Session 1: *n* = 44; Session 2: *n* = 57; Session 3: *n* = 46).

After having assessed the intensity, the participants were asked to take another sniff from the funnels and to answer the following question: “Imagine being exposed to this odor during the day, how acceptable do you think this odor is? Give your opinion with a cross or a dash on the scale below (acceptability)”. For this analysis, the range of the acceptability scale considered is from clearly acceptable = 1 to clearly not acceptable = −1. Table 4 shows the mean values of the acceptability assessment of chambers A and B together with standard deviations (SDs) and standard errors (SEs). Additionally, Figure 9 illustrates that for each of the three sessions, the participants evaluated the air in funnel A less acceptable that the air in funnel B.

Several independent-samples t-tests were conducted to evaluate whether differences between the acceptability assessments of chambers A and B were present during the sessions, and between the sessions. A statistically significant difference between the chambers was found for Session 2 (*p* = 0.045). In general, the participants evaluated the acceptability level of the air in funnels A and B to be less acceptable for each session (Figure 9). For Chamber A, a statistically significant difference for the acceptability of the air assessed in Session 1 and Session 2 (*p* = 0.005) was found, as well as for Session 1 and Session 3 (*p* = 0.012). For Chamber B, no statistically significant differences between the sessions were found.

### 3.4. Odour Recognition

**Figure 10:**
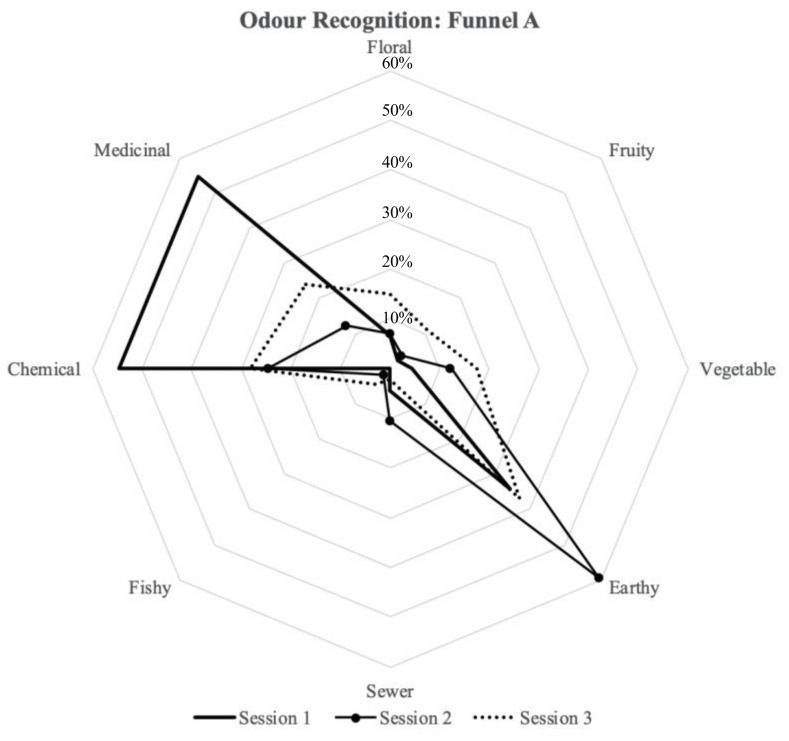
Odor recognition assessment for Chamber A.

**Figure 11:**
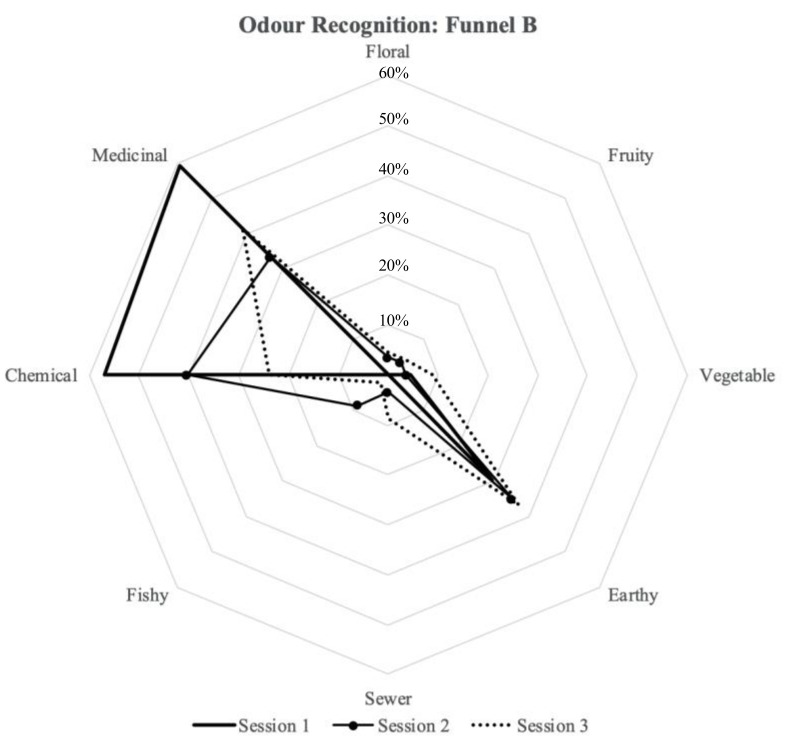
Odor recognition assessment for Chamber B.

The odor acceptability evaluation was followed by an odor recognition test. The participants were asked to take another sniff from the funnels and to answer the following question: “What do you smell? You can choose more than one option (odor recognition)”. Figure 10 and Figure 11 present how the participants identified the odors in funnel A and funnel B. In Session 1, the participants described the odor mainly as medicinal (55%), chemical (55%), and earthy (34%) for Chamber A (furnished with new carpet tiles and with the active plant-based system inside), and medicinal (59%), chemical (57%), and earthy (30%) for Chamber B (furnished with same amount of carpet tiles as Chamber A but without the active plant-based system inside). In Session 2, the participants described the odor mainly as chemical (25%) and earthy (60%) in Chamber A, and earthy (35%), chemical (40%), and medicinal (33%) for Chamber B. In Session 3, the participants described the odor mainly as medicinal (24%), chemical (28%), and earthy (37%) in Chamber A, and medicinal (41%), chemical (24%), and earthy (37%) in Chamber B.

### 3.5. Preference

Last but not least, the participants were asked to answer the following question (about preference): “Which funnel do you prefer?” To evaluate the preference of the participants during the sessions, frequency analysis was performed. Table 5 shows the preference assessment for the two chambers in the three sessions. In Session 1, the participants preferred Chamber A (55%) over Chamber B (45%), while in Session 2 and 3, participants preferred Chamber B over Chamber A.

To evaluate whether there was a statistically significant relation between the starting funnel of the tests and the level of preference of the participants, chi-square tests were performed. The outcome showed no correlation between the starting funnel and the preference level during the three sessions (Session 1: *p* = 0.23; Session 2: *p* = 0.35; Session 3: *p* = 0.57).

## 4. Discussion

### 4.1. Impact of Temperature and Humidity in Human Perception

Previous studies have demonstrated that human sensory evaluation can be used to evaluate the perceived air quality [4,23]. It is well-known that people can use their noses to assess the perceived odor intensity of different materials relatively well and that in general, the equipment that is available is not able to measure the low concentrations of chemical compounds as the nose can [23]. The results of the present study confirmed this: while the chemical measurements showed no VOCs present (emitted from the carpet tiles in the chambers), the participants smelled odors in the air coming out of both of the funnels.

With regards to the physical measurements executed inside of the chambers, the temperature was, in general, always slightly lower in Chamber A, with the plant-based system, than in Chamber B. In contrast, the relative humidity in Chamber A was always higher than in Chamber B, which can be explained by the evaporative cooling effect created by the plant-based system placed in Chamber A [29,30]. During the sessions, the average temperature inside Chamber A was 19.7°C and the average RH was 52%, while in Chamber B, the average temperature was 20.1°C and the average RH was 43%. Taking this into account, it is important to mention that temperature and relative humidity levels of the environment may affect the perceived air quality and could therefore have affected the assessment of the air coming out of the chambers. A lower temperature makes it more difficult to assess the smell [20]. Furthermore, prior findings have shown temperature and humidity have a significant impact on the perception of IAQ. It is stated that the perceived air quality decreases with increasing air temperature and humidity at a constant pollution concentration [31,32].

In this study, the temperature levels in both chambers were rather similar, but the RH levels had a significant difference between the chambers (Table 2). It is important to mention that the outside air coming into both chambers had exactly the same properties, the flooring material in both chambers had the same composition and amount, and in Chamber A, an active plant-based system was place to evaluate its impact in the perceived IAQ. Regarding the RH levels, it is important to mention that any vegetation system generates extra humidity in the environment [33].

### 4.2. Acceptability, Intensity, Odour Recognition, and Preference

The main objective of this study was to test the effect of an active plant-based system on the perceived air quality of a recently furnished room. During the study, the participants were asked to fill in a questionnaire to assess the air coming out of two funnels from two different chambers. The assessment of the two chambers took approximately 5 min per person. This way of assessing was chosen to avoid adaptation to the smell coming out of funnels, since “adaptation” improves acceptability of the air quality [28]. Moreover, the participants were not allowed to enter or see inside of the chambers in order to reduce bias created by an environment that includes the plants [34,35].

The evaluation of air quality expressed in acceptability reflects perceptual information in combination with psychological and social values. The present study showed that the level of acceptability given by the participants in Chamber A, with the plant-based system, was in general lower than in Chamber B. The level of intensity of the odor in Chamber A was evaluated as stronger than in Chamber B. Therefore, when the participants assessed the odor to be more intensive, they also assessed it to be less acceptable. These results are in good agreement with preceding studies [28,36].

With regards to odor recognition, there were three main elements identified by the participants inside of both chambers during the tests: chemical, medicinal, and earthy (Figure 10 and Figure 11). The participants, in general, identified the same elements for the two chambers with slight differences. However, the levels of intensity and acceptability were assessed differently, as shown in Figure 8 and Figure 9. Furthermore, for Sessions 2 and 3, it was seen that the participants preferred Chamber B over Chamber A (Table 5). This can be justified from a psychological point of view: each stimulation introduced in the indoor environment needs justification and explanation; therefore, elements (odors) that are present and that cannot be recognized will lead to some discomfort [37]. Previous studies have shown that perceptual reaction to different odors varies according to individual sensitivity, and in general, when the participants do not know the source of the smell or if they feel that the smell is potentially hazardous, they tend to reject the smell and show their discomfort [38]. Finally, the fact that an active plant-based system was introduced in Chamber A may introduce other pollutants and compounds into the chamber [20], such as mold [33].

### 4.3. Experiments in Semicontrolled Environments

The aim of the study was to evaluate the efficacy of the active plant-based system on the perceived air quality in a semicontrolled environment. To execute this assessment, two test chambers were furnished with the same amount of carpet tiles. Additionally, a plant-based system was placed in Chamber A as presented in Figure 2. Both chambers have the same characteristics and are constructed of low-emitting materials to guarantee a good air quality during the execution of experiments regarding IAQ and IEQ [27].

On one hand, studies have shown that it is “easier” to assess the impact of plant-based systems in terms of IAQ when the experiments are executed in laboratories (closely controlled environments) [14,15] where higher concentrations of pollutants are normally used to evaluate the efficacy of the plants in terms of gaseous pollutant depletion from the air [12,13,14]. On the other hand, in real settings, the concentrations of the gaseous pollutants are lower and diverse, so it is more difficult to assess the efficacy of these systems. Therefore, this study was executed in a test chamber to evaluate the plant-based system in a semicontrolled environment where different features were evaluated in a more “real-setting environment”. However, just one floor material was evaluated, and the participants were not able to interact directly with the prototype, which would be different in a real-setting experiment where many more elements have to be considered and evaluated, such as different materials, different pollutants, and different construction systems. From this study, it is clear that tests in semicontrolled environments are useful to evaluate isolated factors that can improve the removal efficacy of plant-based systems, However, it is also important to evaluate the overall effect of these systems in real settings to understand how these isolated factors will interact with real-setting environments.

### 4.4. Limitations

For this study, the concept of active biofiltration was built in one prototype with a fixed air flow rate created for the fans connected to the plants. It is, therefore, recommended that future studies test the effect of different air flow rates to choose the optimal option. Additionally, Figure 2 and Figure 3 present the setup of the experiment in both chambers, where it is shown that the air coming inside of the chambers is located in the lower part of the wall, directing the air to go over the carpet tiles and not through the system, which can explain the similar assessments of both chambers.

## 5. Conclusions

The aim of the study was to assess the perceived air quality coming out of two different test chambers in the SenseLab: Chamber A, which was furnished with new carpet tiles and an active plant-based system, and Chamber B, which was furnished with the same amount of carpet tiles as in Chamber A, but without the active plant-based system. From the assessments performed in the three sessions, it can be concluded that the level of acceptability in Chamber A was lower than in Chamber B, and the level of intensity was higher in Chamber A than in Chamber B. Moreover, three main odors were identified in both chambers: medicinal, chemical, and earthy. Finally, the participants expressed a slightly higher preference for Chamber B over Chamber A.

Although there are many possible factors that might have influenced the assessments, the outcome indicates that when people do not see the interior details of a room and have to rely on olfactory perception, they prefer a room without plants. The results also show that sensory evaluation is a necessary instrument for the assessment of the indoor air quality because chemical and physical measurements and analysis alone cannot be used in the majority of the cases to predict how the pollutants and their emissions will be perceived by occupants.

Finally, even though previous laboratory studies have shown the chemical depletion of air pollutants in the close surrounding of vegetation [10,11,12,13], from the results reported here, it can be concluded that the presence of this active plant-based system had a slightly negative effect on the perceived air quality.

## Figures and Tables

**Table 1:** Time schedule and assessments.

Session Number	Date	Number of Participants	Assessment			
			Intensity	Acceptability	Recognition	Preference
Pilot Test	4 April 2019	59	x	x	x	-
Session 1	10 April 2019	44	x	x	x	x
Session 2	24 April 2019	57	x	x	x	x
Session 3	8 May 2019	46	x	x	x	x

**Table 2:** Average temperature, relative humidity (RH), and CO_2_ concentration inside of the Chambers A and B during the execution of the sessions.

	Session 1		Session 2		Session 3	
	Chamber A	Chamber B	Chamber A	Chamber B	Chamber A	Chamber B
**Temperature (°C)**	19.5	20.0	19.7	20.2	19.8	20.2
**RH (%)**	38.1	27.3	62.4	54.5	55.5	47.6
**CO_2_ (ppm)**	460.9	262.8	422.5	465.6	480.3	467.4

**Table 3:** Intensity assessment.

	Chamber A			Chamber B		
	Session 1	Session 2	Session 3	Session 1	Session 2	Session 3
**Mean Value**	2.03	2.46	2.5	1.86	1.95	2.30
SD *	0.87	0.94	0.84	0.82	0.88	0.98
SE **	0.13	0.12	0.12	0.12	0.12	0.14

* Standard deviation. ** Standard error.

**Table 4:** Acceptability assessment.

	Chamber A			Chamber B		
	Session 1	Session 2	Session 3	Session 1	Session 2	Session 3
**Mean Value**	0.13	−0.11	−0.11	0.19	0.09	0.01
SD *	0.38	0.56	0.51	0.37	0.49	0.54
SE **	0.06	0.08	0.07	0.06	0.06	0.08

* Standard deviation. ** Standard error

**Table 5:** Preference assessment for the two chambers in the three sessions.

Preference	Session 1		Session 2		Session 3	
	*n* = 44		*n* = 57		*n* = 46	
**Funnel 1 (A)**	24	55%	21	37%	17	37%
**Funnel 2 (B)**	20	45%	35	61%	28	61%
**No preference**			1	2%	1	2%
**Total**	44	100%	57	100%	46	100%

## Data Availability

More information on the data presented in this study is available on request from the corresponding author. The data are not publicly available due to restrictions regarding the privacy of the participants.
